# A Dimerized HMX1 Inhibits *EPHA6*/*epha4b* in Mouse and Zebrafish Retinas

**DOI:** 10.1371/journal.pone.0100096

**Published:** 2014-06-19

**Authors:** Fabienne Marcelli, Gaëlle Boisset, Daniel F. Schorderet

**Affiliations:** 1 IRO – Institute for Research in Ophthalmology, Sion, Switzerland; 2 Faculty of Life Sciences, Swiss Federal Institute of Technology (EPFL), Lausanne, Switzerland; 3 Faculty of Biology and Medicine, University of Lausanne, Lausanne, Switzerland; Institut Curie, France

## Abstract

*HMX1* is a homeobox-containing transcription factor implicated in eye development and responsible for the oculo-auricular syndrome of Schorderet-Munier-Franceschetti. *HMX1* is composed of two exons with three conserved domains in exon 2, a homeobox and two domains called SD1 and SD2. The function of the latter two domains remains unknown. During retinal development, HMX1 is expressed in a polarized manner and thus seems to play a role in the establishment of retinal polarity although its exact role and mode of action in eye development are unknown. Here, we demonstrated that HMX1 dimerized and that the SD1 and homeodomains are required for this function. In addition, we showed that proper nuclear localization requires the presence of the homeodomain. We also identified that *EPHA6*, a gene implicated in retinal axon guidance, is one of its targets in eye development and showed that a dimerized HMX1 is needed to inhibit *EPHA6* expression.

## Introduction

Homeobox-containing transcription factors represent an important class of factors involved in the regulation of embryogenesis and other molecular programs. *HMX1* is a homeobox-containing transcription factor implicated in eye development. In 1992, Stadler et al. described a new homeobox gene called *GH6*. This gene was later renamed *HMX1* and was assigned to the NKX5 family, the reason why *HMX1* is also known as *NKX5-3* [1]. Later, further members were identified: *HMX2* (*NKX5-2*), *HMX3* (*NKX5-1*) and, in chicken, zebrafish and medaka, *SOHo-1* [2]–[4]. The NKX5/HMX family of transcription factors contains a unique homeobox region that is phylogenetically conserved. *HMX1*, *HMX2* and *HMX3* contain two other conserved domains called SD1 and SD2, located immediately C-terminally to the homeobox [5]. The function of these domains is still unknown.

Whereas Hmx2 and Hmx3 play a role in inner ear development, Hmx1 and SOHo-1 are mainly implicated in eye development. In the mouse eye, *Hmx1* expression can be detected as early as E10.5, and transcripts are more specifically present in the lens and in the antero-medial part of the neural retina [4]–[8]. In the developing chicken eye, it is expressed in the dorsal neural retina and lens epithelium as well as in the optic nerve [9]. *HMX1* expression starts 40 hours into development (stage 11) in the surface ectoderm surrounding the optic vesicle. At optic cup invagination (stage 14–15), it is expressed in the anterior/nasal side of the early retina [10]. In zebrafish, *hmx1* is first expressed in the entire eye at 10 somite-of-stage (ss), and is then repressed in the dorsal part at 18 ss. At 24 hours post fertilization (hpf), it is restricted to the nasal retina and, one day later, expression is restricted to the nasal part of the ganglion cell layer (GCL). At four and five days post fertilization, signal is also observed in the nasal part of the inner nuclear layer (INL). In the developing lens, expression is observed from 24 to 72 hpf [11], [12].

We recently reported a family with a 26-bp deletion in exon 1 of *HMX1* leading to the oculo-auricular syndrome of Schorderet-Munier-Franceschetti (OMIM: 612109), characterized by microphthalmia, microcornea, nystagmus, cataract, coloboma, optic nerve dysplasia, RPE abnormalities, rod-cone dystrophy and deformation of the ear lobule [12], [13]. A mouse model containing a mutation in *Hmx1* has been described [6]. It shows laterally protruding ears, subtle changes in cranial bone morphology, perinatal semi-lethality, reduced body mass and microphthalmia with low-grade keratoconjunctivitis sicca and entropion. The eyes show no evidence of microcornea, anterior segment dysgenesis, cataract, coloboma, retinal detachment or retinal dysplasia. Quina et al. observed a significant reduction of geniculate ganglion neurons [7]. *In vitro*, HMX1 binds to a 5′-CAAGTG-3′ sequence, represses transcription from a luciferase reporter containing this binding site and can antagonizeNKX2.5, a cardiac homeo protein, which is activating this same reporter construct [14]. Nkx2.5 is also known to dimerize at its homeodomain and other regions in the C-terminus [15].

In this study, we showed that HMX1 acted as a dimer and that the homeobox and the conserved domain SD1 were needed for dimerization to occur. SD2 was not involved in the dimerization process. We also identified *EPHA6* as a target of HMX1 and showed that HMX1 repressed the *EPHA6* promoter *in vitro*. The inhibitory activity of HMX1 was associated with the presence of the SD1 and homeobox domains. Whereas the *EPHA6* inhibition was lost with mutants of each of these 2 domains, the SD2 mutant showed a small activation of the *EPHA6* promoter. Mutation of the three CAAG(TG) sequences of the promoter attenuated the repression by HMX1. This inhibition was confirmed *in vivo* in zebrafish embryos.

## Materials and Methods

### Plasmid Constructions

Subcloning was performed according to standard protocols. Mutagenesis was performed using the QuickChange II Site-Directed Mutagenesis Kit (Stratagene, Agilent Technology AG, Basel, Switzerland). The sequence of the primers used in this study is available from the authors.

### Cell Culture and Transfection

Human embryonic kidney (HEK) 293T cells were cultured at 37°C and in 5% CO_2_ atmosphere, in Dulbecco’s Modified Eagle’s Medium (DMEM) high glucose with stable glutamine (GE-Healthcare, Glattbrugg, Switzerland), supplemented with 10% FBS (Lonza, Basel, Switzerland), 100 U/ml penicillin and 100 µg/ml streptomycin (Invitrogen, Basel, Switzerland). Transfection was performed using the Calcium Phosphate method (ProFection Mammalian Transfection System, Promega, Dubendorf, Switzerland).

### BRET^2^

200’000 HEK 293T cells in DPBS were distributed into black 96-well microplates for fluorescence quantification. Filter sets were adapted to 485 nm for GFP^2^ excitation and 510 nm for emission. Cells expressing BRET^2^ donor (RLUC) alone were used to determine the fluorescence background. 200’000 cells with comparable fluorescence levels were distributed into white 96-well microplates for luminescence quantification. The luciferase substrate Coelenterazine 400A, DeepBlueC (Chemie Brunschwig, Basel, Switzerland) was added to a final concentration of 5 µM. Filter sets were adapted to 410 nm for luciferase emission and 515 nm for GFP^2^ emission. The emitted fluorescence and luminescence were measured using an Envision 2103 Multilabel Reader (PerkinElmer, Schwerzenbach, Switzerland), and analyzed with the Wallac Envision Manager V1.12 software (PerkinElmer, Schwerzenbach, Switzerland).

### Co-immunoprecipitation

200 µg of proteins were immunoprecipitated overnight at 4°C on a rotating wheel with 2.5 µl anti-Renilla Luciferase antibody (MAB4400, Millipore, Zug, Switzerland). 20 µl of washed protein G plus agarose beads (Santa Cruz, LabForce AG, Nunningen, Switzerland) were added and incubated 2 hrs at 4°C on the rotating wheel. After centrifugation at 4°C, the supernatants were kept as controls. The pellets were resuspended in 25 µl 2x SDS loading buffer and loaded on a 12% SDS-page gel, alongside with 20 µl of supernatant and 40 µg of proteins.

### Western Blot

Proteins were extracted from cell cultures using RIPA (50 mM Tris-HCl pH 8.0, 150 mM NaCl, 1% NP-40, 0.5% sodium deoxycholate, 0.1% SDS) and concentrations measured using the Micro BCA Protein Assay Kit (Thermo Fisher Scientific, Reinach, Switzerland) on a Multiplate Reader Synergy HT (Bio-Tek, Luzern, Switzerland) with the KC4 software. The following antibodies were used: HA-Tag (6E2) Mouse mAb #2367 (Cell Signaling, LabForce AG, Nunningen, Switzerland), GFP N-terminal G1544 (Sigma, Buchs, Switzerland), PARP (46D11) Rabbit mAb #9532 (Cell Signaling, Labforce AG, Nunningen, Switzerland), p62/SQSTM1 P0067 (Sigma, Buchs, Switzerland), Ub (A-5) sc-166553 (Santa Cruz Biotechnology, LabForce AG, Nunningen, Switzerland) and α-Tubulin Clone B-5-1-2 T5168 (Sigma, Buchs, Switzerland).

### Native Western Blot

Cells were lysed in a non-denaturing lysis buffer (20 mM Tris-HCl, 137 mM NaCl, 10% glycerol, 1% Triton X-100, 2 mM EDTA, pH 8.0). The protein concentrations were measured as described above. 5 µg were loaded on a Mini-Protean TGX precast gel 4–15% (BioRad Laboratories AG, Cressier, Switzerland) with non-denaturing loading buffer (300 mM Tris-HCl pH 7.8, 30% glycerol, 0.6% bromophenol blue) and migrated without denaturation in a running buffer without SDS.

### GFP^2^ Fluorescence Imaging and Nuclei Isolation

Cells were analyzed 48 hrs post-transfection under a Zeiss Axiovert 200 microscope with filters adapted for excitation and emission at λex = 480 nm and λem = 510 nm, respectively, and the AxioVision 4.2 software. For nuclei isolation, cells were counted and resuspended at 10^8^ cells/ml Nuclei Isolation Buffer (250 mM sucrose, 20 mM Hepes pH 7.8, 10 mM KCl, 1.5 mM MgCl_2_, 0.5 mM spermidin). Cells were then homogenized with a Potter and spread on a slide.

### In Silico Search for a Nuclear Localization Signal (NLS)

The mouse HMX1 sequence was entered in the NLS-Mapper software that can be found at http://nls-mapper.iab.keio.ac.jp/cgi-bin/NLS_Mapper_form.cgi.

### Immunofluorescence

Immunofluorescence was performed 24 hrs post transfection. When necessary, 50 µM chloroquine were added for 16 hrs. The primary antibody (LC3B #2775, Cell Signaling, LabForce AG, Nunningen, Switzerland) was diluted in 1x PBS +2% NGS +0.2% Triton X-100 and incubated overnight at 4°C in a humid chamber. The secondary antibody (Alexa Fluor 594 goat α-rabbit IgG (H+L) (A11012), Molecular Probes, LubioScience, Luzern, Switzerland) was diluted in the same buffer, and incubated 1 hr at RT in a humid chamber in the dark. Nucleic acids were stained with 100 µM DAPI (4,6-diamidino-2-phenyl-indole HCl) (1/1′500 in 1x PBS) for 10 min in a humid chamber in the dark. Cells were then mounted with Citifluor AF1 (Citifluor Ltd, Leicester, UK), and conserved at 4°C. The slides were analyzed under an Olympus BX61 microscope and the Cell^M^ software (Olympus, Volketswil, Switzerland).

### Hoechst-PI Staining

20 mg/ml bisBenzimide H 33342 trihydrochloride (Sigma, Buchs, Switzerland) and 1 mg/ml Propidium Iodide (Fluka, Buchs, Switzerland) were diluted 1/2′000 into the culture medium. Cells were analyzed under a Zeiss Axiovert 200 microscope and the AxioVision 4.2 software.

### Luciferase Assays

48 hrs post transfection cells were washed with 1x PBS, and 300 µl luciferase assay lysis buffer (100 mM K_2_HPO_4_ pH 7.8, 0.2% Triton X-100) were added. Cells were scraped at 4°C and centrifuged 3 min at 12′000 rpm at 4°C. 5 µl of supernatant were transferred to a transparent 96-well plate containing 50 µl 2x β-gal buffer (120 mM Na_2_HPO_4_, 80 mM NaH_2_PO_4_, 2 mM MgCl_2_, 100 mM β-mercaptoethanol). 50 µl of 2x ONPG (1.33 mg/ml 2-nitrophenyl-B-D-galactopyranoside) were added and the plate read at 412 nm of absorbance on a Multiplate Reader Synergy HT (Bio-Tek, Luzern, Switzerland) with KC4 software. If the values were constant in all conditions, 5 µl of supernatants were transferred to a white 384-well plate, 20 µl of Luciferase Assay Reagent (Promega, Dubendorf, Switzerland) were added and luminescence measured on the Multiplate Reader Synergy HT every 3 minutes until the peak of luciferase activity was reached. The obtained values were normalized using a β-gal reporter under the control of a CMV promoter. A mean between the 3 highest values was used for the luciferase/β-gal ratio. Each experiment was performed three times in duplicates. Only transfections with stable β-gal values between the different conditions, indicating similar transfection efficiency, were used. Two-tailed Student’s T-tests with unequal variance were used to determine statistical differences between the conditions.

### Chromatin Immunoprecipitation

All experiments involving live animals were authorized by the Veterinary Service of the State of Valais under authorizations N° VS-13 and VS-19. The litter of four was housed with the mother and was anesthetized with isoflurane prior to being euthanized by cervical dislocation. Retinas from four 2-week-old wild-type C57Bl/6J mice were dissected, fixed, and homogenized. Glycine was added to a final concentration of 0.125 M before centrifugation. The pellet was resuspended in nuclei lysis buffer (50 mM Tris-HCl pH 8.0, 10 mM EDTA pH 8.0, 1% SDS). The resulting chromatin was sonicated, snap-frozen in liquid nitrogen, and kept at −80°C. The next day, the tube was centrifuged, and the supernatant transferred to new eppendorf tubes containing 10 µl of Protein A-Agarose beads (Roche, Basel, Switzerland). 100 µl of supernatant were pre-cleared with the 10 µl of beads for 1 hr on a rotating wheel at 4°C. The tubes were centrifuged and the supernatants transferred to new tubes. A control tube containing 10 µl 5% BSA and a test tube with 2 µl Hmx1 antibody (ARP32629_P050, Aviva, LubioScience, Luzern, Switzerland) were prepared. The tubes were incubated overnight at 4°C on a rotating wheel. After 10 µl of Protein A-Agarose beads were added to both tubes and another incubation, the tubes were centrifuged and the supernatants were kept at −80°C ( = TIC). The pellets were washed in IP wash buffer n°1 (0.1% SDS, 1% Triton X-100, 20 mM EDTA pH 8.0, 150 mM NaCl, 20 mM Tris-HCl pH 8.0), 4 times in IP wash buffer n°2 (0.1% SDS, 1% Triton X-100, 2 mM EDTA pH 8.0, 500 mM NaCl, 20 mM Tris-HCl pH 8.0), once in IP wash buffer n°3 (250 mM LiCl, 1% NP-40, 1% deoxycholate, 1 mM EDTA pH 8.0, 10 mM Tris-HCl pH 8.0) and once in TE 10∶1 (10 mM Tris-HCl pH 7.5), 1 mM EDTA pH 8.0). The antibody was eluted from beads by adding 150 µl IP elution buffer (50 mM NaHCO_3_, 1% SDS) twice and by shaking 15 min at RT. 12 µl 5M NaCl and 1 µl RNase A (10 mg/ml) (Roche, Basel, Switzerland) were added and the tubes were incubated 5 hrs at 67°C. The TIC samples were thawed and 100 µl transferred to new tubes. 500 µl IP elution buffer, 24 µl 5 M NaCl and 2 µl RNase A (10 mg/ml) were added, and the tubes were incubated 5 hrs at 67°C. After incubation, 2.5 volumes 100% EtOH were added for precipitation overnight at 4°C. The next day, the tubes were centrifuged and the pellets were dissolved in 100 µl TE 10∶1. 25 µl proteinase K buffer for ChIP (50 mM Tris-HCl pH 7.5, 25 mM EDTA pH 8.0, 1.25% SDS) and 1 µl proteinase K (Roche, Basel, Switzerland) were added, and the tubes incubated 2 hrs at 45°C. 175 µl TE 10∶1 and 300 µl Phenol:Chlorophorm:Isoamyl Alcohol 25∶24∶1 (Sigma, Buchs, Switzerland) were added, the tubes shaken and centrifuged. 30 µl of 5 M NaCl, 1 µl of 5 mg/ml glycogen and 750 µl of 100% EtOH were added to the supernatants, mixed and precipitated overnight at 4°C. The next day, the tubes were centrifuged, the supernatants were removed and the pellets resuspended in 30 µl TE 10∶1. PCR analysis was performed on 2 µl of samples.

### Generation of the Zebrafish Hsp70-HMX1 Transgenic Line

AB zebrafish were raised and kept under standard laboratory conditions at 28.5°C. Transgenesis was performed by generating Tol2 transposon constructs using the tol2kit [16]. The zebrafish *hmx1* coding sequence was cloned downstream of the hsp70 promoter and the DNA construct together with the transposase mRNA were injected at the one-cell stage. Fish were raised to adulthood and the cardiac GFP expression was used as a marker for germline transmission. Experiments were done on F3 obtained from F2 that were intercrossed in order to increase the number of larvae carrying the transgene. Tg (hsp70: hmx1) and wt were heat shocked at 1 dpf during 30 min at 39°C, euthanized and fixed 4 hrs after.

### Whole-mount *in situ* Hybridization

Standard one-color whole-mount *in situ* hybridization was performed at various stages. Hybridization reaction was done at 68°C for 14–18 hrs. Washing steps and antibody incubation were performed in an *in situ* machine (BioLane HTI, Hölle&Hüttner, Tubingen, Germany). Templates used to generate DIG-labeled RNA probes included zebrafish *hmx1* (ID: 797503), *epha4b* (ID: 64270) and *pax6* (ID: 60639). *In vitro* transcription was done with the Roche RNA Labeling Kit (Roche Applied Science, Basel, Switzerland).

## Results

### HMX1 dimerizes through the SD1 and homeobox domains

The ability to homo- or heterodimerize has been demonstrated for many transcription factors, including the NKX member NKX2-5, a cardiac homeobox gene that dimerizes through its homeodomain (HD) [15]. We therefore investigated, using a BRET^2^ approach, whether HMX1 behaved similarly and formed dimers or oligomers. The BRET^2^ technique is based on the energy transfer occurring between the renilla luciferase (RLUC) and the green fluorescent protein (GFP) when they are in close proximity. The principle of the technique is to generate fusion proteins between proteins of interest and the RLUC and the GFP, and measure the energy transfer in culture conditions to determine if the proteins of interest are interacting.

**Figure 1:**
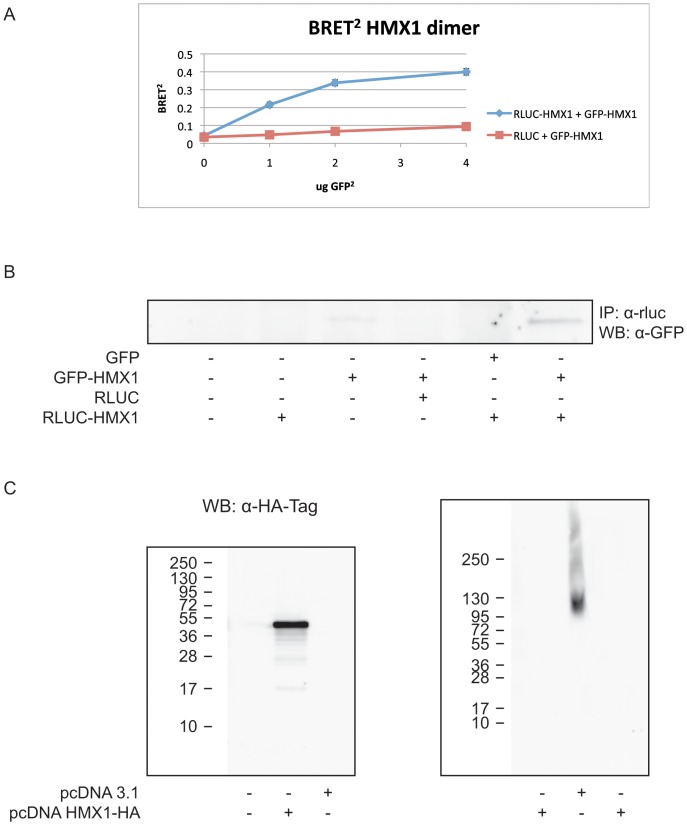
Dimerization of HMX1 in HEK 293T cells. HMX1 is dimerizing as shown by the increasing BRET^2^ ratio in presence of the two fusion proteins. Data points represent the mean of two experiments +/− SD (A). Co-immunoprecipitation was only observed in presence of the two HMX1 fusion proteins. IP was performed with anti-Renilla Luciferase antibody and WB with anti-GFP antibody (B). The size of the HMX1 band revealed with anti-HA-Tag (6E2) Mouse mAb was twice the size in non-denaturing conditions as in denaturing conditions. As no higher molecular weight bands were observed, it is unlikely that trimers were formed (C).

When plasmids expressing fusion proteins between RLUC and HMX1, and GFP^2^ and HMX1 were mixed and transfected in HEK 293T cells, a robust increase in the BRET^2^ ratio was observed with increasing concentrations of GFP^2^-HMX1, indicating that HMX1 dimerized (Figure 1A). We confirmed dimerization of HMX1 by co-immunoprecipitation (co-IP) using an RLUC antibody for immunoprecipitation and a GFP antibody for blotting. Out of the six conditions tested, the only condition in which immunoprecipitation occurred was when the two different HMX1 fusion proteins were present (Figure 1B). Non-denaturing electrophoresis was also used to further confirm this result. As no western-blot suitable antibody against HMX1 existed, we tagged HMX1 with an HA-tag and used antibodies against HA to visualize the fused HA-HMX1 protein. HA-tagged wild-type HMX1 proteins were loaded on a non-denaturing native electrophoresis gel and sizes were compared to a denaturing gel after western blot analysis with an anti-HA antibody. The size of the band in non-denaturing conditions was twice the size of the band in denaturing conditions, suggesting dimerization. As no bands of higher molecular weight were observed, it is unlikely that trimers or other multimers were formed (Figure 1C).

**Figure 2:**
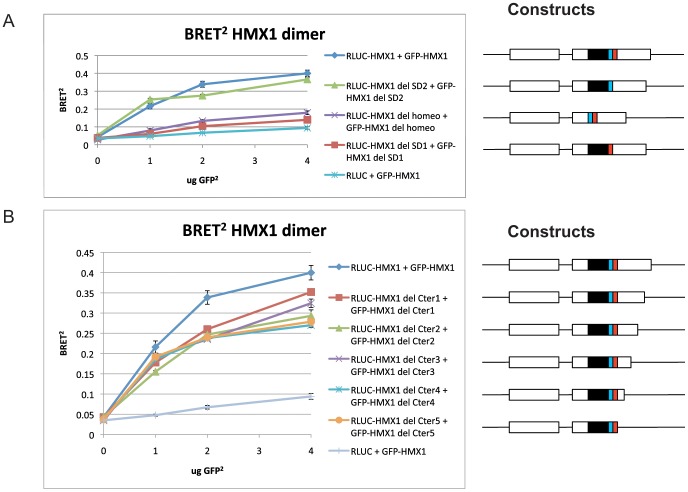
Identification of the domains of HMX1 needed for dimerization. Deletions of two of the conserved domains, the homeobox (black) and SD1 (blue) prevented dimerization. Deletion of the SD2 domain (red) had no effect (A). Deletion of the C-terminus of the protein does not prevent dimerization (B). Data points represent the mean of two experiments +/− SD.

In order to determine the dimerization domain of HMX1, we generated deletions of various portions of the protein. *HMX1* is composed of two exons, with three conserved domains in exon two: the homeobox (HD), and two domains called SD1 and SD2, located 3′ to the HD and whose function is presently unknown. We deleted each of these domains separately by site-directed mutagenesis and repeated the BRET^2^ experiments. As shown in figure 2A, deletions of HD or SD1 led to the loss of dimerization, whereas deletion of SD2 had no effect. This suggested that HD and SD1 were implicated in the dimerization of HMX1. In order to confirm these results and to show that the HMX1 C-terminal region was not involved in dimerization, in contrary to that of NKX2-5, we generated serial deletions of the C-terminal part of the protein. None of these constructs prevented dimerization as shown by BRET^2^ (Figure 2B).

### The Entire HD is Necessary for Correct Nuclear Localization of HMX1

**Figure 3:**
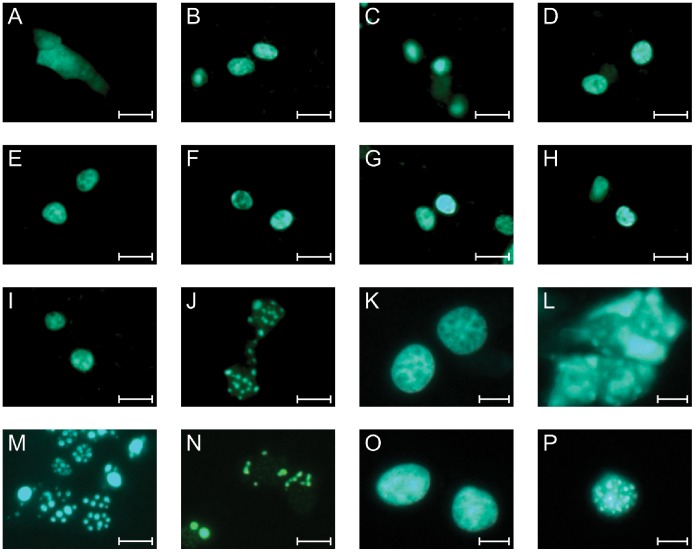
Cellular expression of the fusion proteins between GFP and HMX1 and its mutants in HEK 293T cells. GFP is expressed in the whole cell (A), whereas GFP-HMX1 is expressed in the nucleus (B). The different mutants, GFP-HMX1 del SD1 (C), GFP-HMX1 del SD2 (D), GFP-HMX1 del Cter1 (E), GFP-HMX1 del Cter2 (F), GFP-HMX1 del Cter3 (G), GFP-HMX1 del Cter4 (H) and GFP-HMX1 del Cter5 (I) retain a nuclear localization. GFP-HMX1 del HD on the other hand is expressed in a punctate manner (J). These aggregates are present in the nucleus as well as in the cytoplasm: compare L (GFP-HMX1 del HD before nuclei isolation) and P (GFP-HMX1 del HD after nuclei isolation) to K (GFP-HMX1 before nuclei isolation) and O (GFP-HMX1 after nuclei isolation). Reinsertion of the seven-amino acid KKTRTVF did not change the punctate phenotype (M), nor did the deletion of the Cter half of the homeobox (N). Scale bars: 50 µm for A-J, M, N; 10 µm for K, L, O, P.

Fusing HMX1 to the GFP^2^ reporter allowed us to visualize its cellular localization. GFP alone localized to the cytoplasm (Figure 3A), whereas GFP-HMX1 localized to the nucleus (Figure 3B). All of the generated mutants retained this nuclear localization except one (Figure 3C–J). The HD deletion mutant was expressed in a punctate manner in the nucleus as well as in the cytoplasm (Figure 3K, L, O, P). This punctate phenotype could possibly be due to the loss of a nuclear localization signal located in the homeobox. We therefore tested the sequence for potential nuclear localization signals (NLS) using NLS mapper, a bioinformatic tool available online. The analysis of HMX1 revealed the presence of a monopartite NLS -RGGRRKKTRTVF-, with KKTRTVF corresponding to the very beginning of the HD, with a score of 9.5. Deleting this signal could thus explain why the GFP-HMX1 del HD protein lost its nuclear localization. To test this hypothesis, we reinserted the seven-amino acid KKTRTVF into the HMX1 del HD sequence. However, reinserting these amino acids did not modify the punctate expression and localization of this mutant (Figure 3M). To verify if the predicted NLS needed additional amino acids to be functional, we generated a new mutant with a deletion of the C-terminal half of HD (30 amino acids). However, this construct was still expressed in a punctate manner similar to the deletion of the entire HD (Figure 3N).

**Figure 4:**
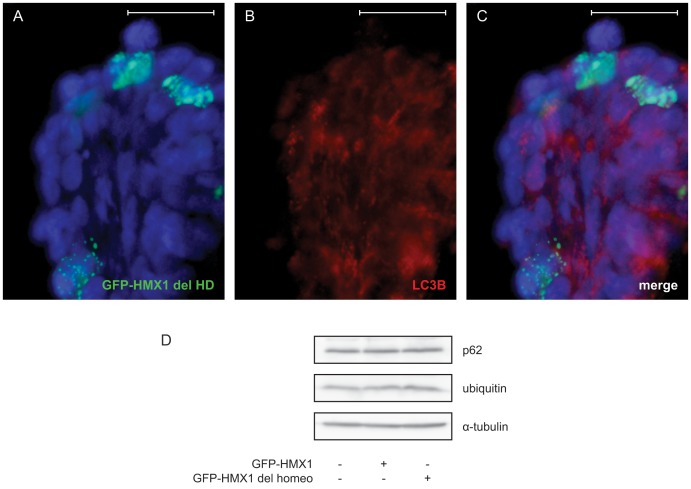
Determination of the nature of the GFP-HMX1 del HD aggregates. GFP-HMX1 del HD aggregates do not colocalize with LC3B and are thus not included in autophagosomes (A–C). Cells expressing GFP-HMX1 del HD (green) present no increased autophagy, and HMX1 del HD aggregates do not colocalize with autophagosomes (LC3B staining in red, after 50 µM chloroquine treatment for 16 hrs). Likewise, p62 and ubiquitin levels were not increased, confirming that autophagy was not activated (D). Scale bars: 50 µm.

The homeobox of HMX1 is of helix-turn-helix-loop-helix type. It is likely that removal of any part of this structure prevents the correct folding of the protein and that it activates clearance mechanisms. In an effort to determine the nature of the aggregates generated by the GFP-HMX1 del HD mutant, we tested several hypotheses. First, the shape, size and distribution of the aggregates suggested that they could be autophagosomes induced by the abundant expression of aberrant proteins. We therefore verified if GFP-HMX1 del HD colocalized with LC3B by immunofluorescence, but this was not the case (Figure 4A, B, C). Even after treating cells with chloroquine to visualize autophagosomes, GFP-HMX1 del HD did not colocalize with autophagosomes. To confirm this result, we also tested whether *p62* and *ubiquitin* expression was increased in the presence of GFP-HMX1 del HD. The role of ubiquitin is to clear abnormal proteins by targeting them for degradation by the 26S proteasome. Poly-ubiquitinated protein aggregates are also sequestered in inclusion bodies containing p62, and the aggregates are cleared via autophagy. In our experiments, we did not observe any increase in expression of these two proteins, indicating that these mechanisms were not activated (Figure 4D). To determine whether the cells were suffering from the presence of GFP-HMX1 del HD aggregates, we looked for the presence of increased apoptosis by PARP cleavage assay. Cleavage of PARP by Caspase-3 is a step in the cascade leading to apoptosis. However, we failed to show any such increase (data not shown). Moreover, no increased cell death was observed when performing a Hoechst-PI staining for dying cells (data not shown). The exact nature of these punctae could thus not be determined and we do not know at this time whether they represent pure HMX1 aggregates or a more complex structure.

### HMX1 Binds to the Promoter of *EPHA6/epha4b* and Inhibits its Expression

HMX1 and SOHO-1 are defining the EPHA3 expression domain in the developing chick retina [10]. Ephrins act as topographically specific repulsive guidance cues for ganglion cell axons. EPHA3 is expressed in a temporal>nasal gradient in the developing chick retina and is present on ganglion cell axons during the time of target innervations. HMX1 and SOHO-1 are expressed in an inversed gradient to that of EPHA3 (nasal>temporal), and when HMX1 and SOHO-1 are expressed ectopically, EPHA3 expression is lost. *EPHA3* thus appeared to be a good candidate as a target for HMX1. However, ephrins do not have the same patterns of expression and do not play the same roles between different species. In the ganglion cell layer, where Hmx1 is expressed on the nasal side, EphA5 and EphA6 (P0 mouse) and EPHA3 (chicken) are only expressed on the temporal side [10], [17], [18]. Chicken EphA5 and EphA6 are uniformly expressed in the chick retina, and EphA3 is not expressed in the mouse retina GCL [17], [19]–[21]. Therefore mouse EphA5 and EphA6 seem to be functional homologs of chicken EPHA3, which suggests that HMX1 could repress the activity of the EphA5 or EphA6 promoter in mouse. In zebrafish, epha4b is expressed in the same temporal pattern as chicken EPHA3 and mouse EphA5 and EphA6, whereas epha6 is not expressed in the eye (not shown).

**Figure 5:**
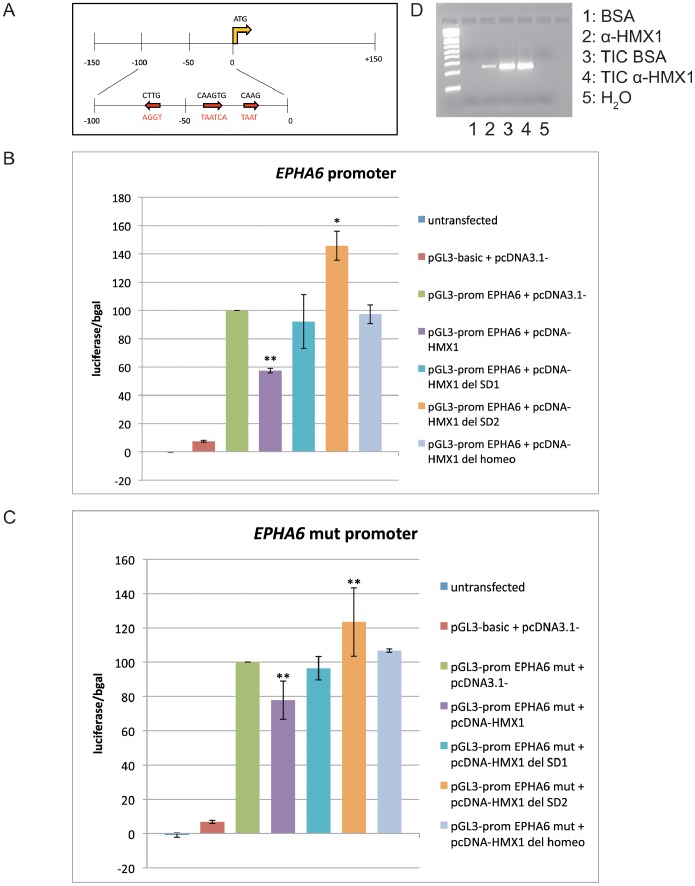
Action of HMX1 on the *EPHA6* promoter. Schematic representation of the subcloned fragment of the *EPHA6* promoter with the three binding sites (red arrows and black characters), and the mutated sequences (red characters) (A). Luciferase assay on the wt *EPHA6* promoter with HMX1, HMX1 del SD1, HMX1 del SD2 and HMX1 del HD. HMX1 inhibits the promoter by 42%, HMX1 del SD1 and HMX1 del HD have no effect, and HMX1 del SD2 slightly activates the promoter (B). Chromatin immunoprecipitation on 2-week-old C57Bl/6J retinas demonstrated the physical interaction between HMX1 and the *EphA6* promoter. 5% BSA was added in the control conditions instead of the Hmx1 antibody. TIC = total input chromatin (C). Mutation of the *HMX1* binding sites attenuates the effect of HMX1 and HMX1 del SD2 but does not completely abolish it (D). Data points represent the mean of three experiments +/− SD. ** : P<0.01. * : P<0.05 (Student’s T-test).

**Figure 6:**
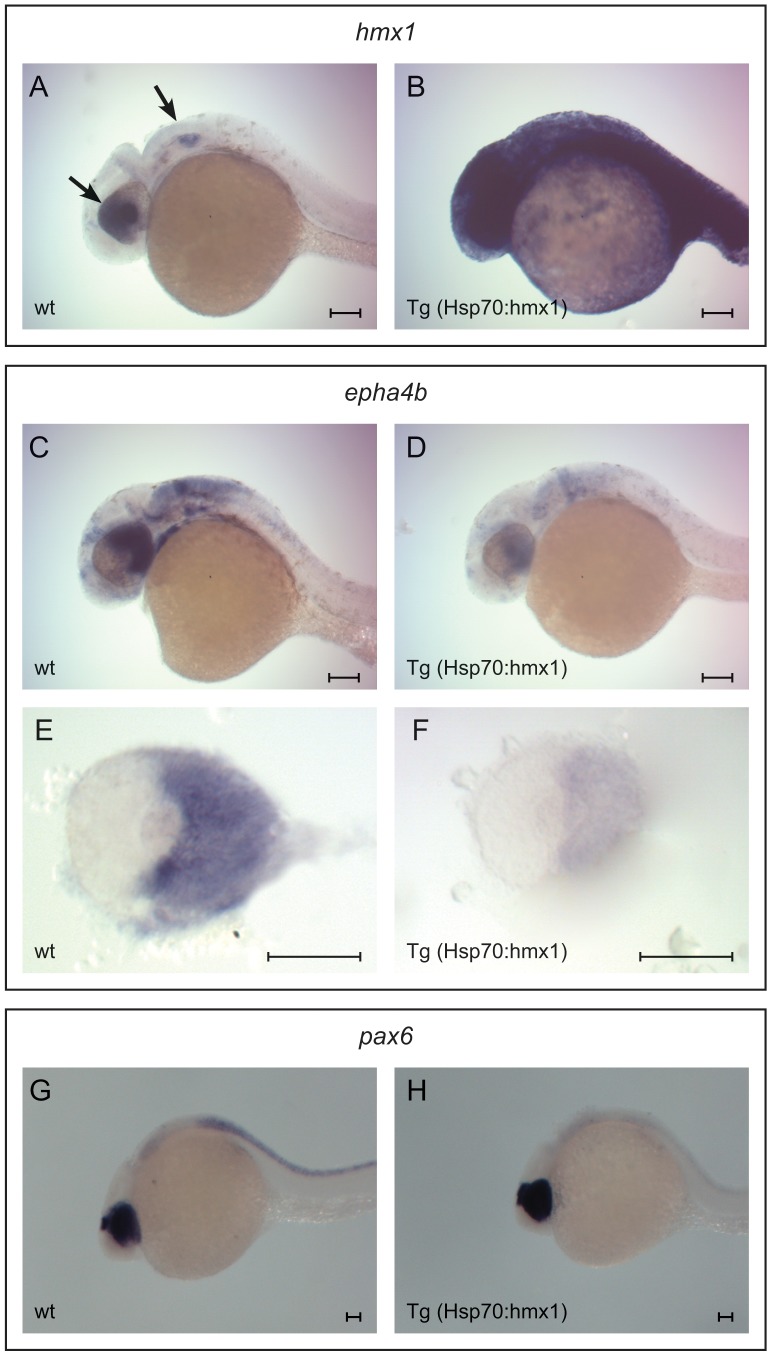
Regulation after *hmx1* misexpression in zebrafish. Expression of *hmx1* after heat shock in wt and tg (hsp70:hmx1) embryos (A, B). *Hmx1*, normally restricted to the nasal retina, lens and ear (arrows in A), was broadly expressed in the transgenic embryo (B). *Epha4b* expression after heat shock in wt and Tg (hsp70:hmx1) embryos (C–F). Dissected eye showed a strong reduction of *epha4b* expression in the temporal retina when *HMX1* was co-expressed (E, F). *Pax6* regulation after *HMX1* misexpression in zebrafish. Ocular expression of *pax6* in wt (G) was not modified by overexpression of *HMX1* in Tg(hsp70:hmx1) embryos (H). Scale bars: 100 µm.

Amendt et al. showed that HMX1 was preferentially binding to a CAAG(TG) sequence [14]. The *EPHA6* promoter contains three such binding sites, the second being conserved between human and mouse (−39 relative to the ATG), whereas the *EPHA5* promoter does not contain any. *EPHA6* was also identified as a potential target of HMX1 using a predictive promoter model that we recently developed [22]. We therefore analyzed the effect of HMX1 on the human *EPHA6* promoter. The technique we used allowed measuring the activity of the promoter by placing a luciferase reporter under its control. We subcloned a fragment spanning from −150 to +150 nucleotides relative to the *EPHA6* translation initiation codon into a luciferase reporter vector. This fragment represented the minimal *EPHA6* promoter with a 13-fold increased activity compared to pGL3-basic vector. Shorter fragments (−100 to +150 and −50 to +150) displayed reduced promoter activity (five- and three-fold increased activity, respectively, compared to pGL3-basic vector) whereas the +1 to +150 fragment displayed no promoter activity (data not shown). The measured activity values were normalized using a β-gal reporter under the control of a CMV promoter. The subcloned fragment contained three potential binding sites for HMX1: one CAAGTG in the forward direction at position –39, one CAAG in the forward direction (−20) and one CAAG in the reverse direction (−65) (Figure 5A). We performed luciferase assays with wild-type HMX1 and the mutants deleting the HD, SD1 or SD2. As shown in figure 5B, HMX1 was inhibiting the *EPHA6* promoter activity by 42%. The physical interaction between HMX1 and the *EphA6* promoter was demonstrated by chromatin immunoprecipitation on retinas isolated from two-week-old C57BL/6J mice (Figure 5C), a technique allowing to determine which proteins bind to a DNA fragment by crosslinking them and selecting for the fragments bound to the protein by immunoprecipitation and PCR amplification of the fragment. We also validated this interaction *in vivo* on the zebrafish *epha4b* gene, the functional homolog of *EPHA6*, having two HMX1 binding sites in its promoter. We generated a transgenic fish line expressing a ubiquitous heat-shock activated *hmx1* gene and showed by *in situ* analysis that the aberrant ectopic expression of hmx1 in the temporal retina reduced the expression of *epha4b* (Figure 6A–F), which was not the case for the control gene *pax6* (Figure 6G–H).

We then checked whether dimerization was needed for HMX1 repressive activity. Mutant constructs preventing dimerization, i.e. deletions of HD or SD1, had no activity, indicating that only dimerized HMX1 regulates *EPHA6* expression. The mutant with a deleted SD2 domain slightly activated the *EPHA6* promoter, suggesting that this region might represent the binding site of a cofactor needed for the inhibitory activity of HMX1 (Figure 5B). In order to confirm that the CAAG/CAAGTG sites represented *bona fide* binding sites for HMX1, we mutated them into the sequences shown in red in figure 5D. Control experiments showed that these mutations did not affect *EPHA6* promoter activity (data not shown). Transfection experiments using these mutated constructs showed a reduction of the inhibitory activity of HMX1 from 42% to 22% (Figure 5D). This indicates that the CAAG/CAAGTG sites represent true binding sites for HMX1. However additional sites might exist, as deletion of CAAG/CAAGTG sites failed to completely abrogate the inhibition.

## Discussion

The interest in the HMX1 transcription factor has surged with the discovery in 2008 that it was causing the oculo-auricular syndrome of Schorderet-Munier-Franceschetti [12]. In addition of being expressed in somatosensory organs [23], Hmx1 has been shown to retain a neuronal fate in migrating neural crest cells [24] and to modulate the adrenergic/cholinergic program of sympathic neurons [25]. It is also well expressed in sensory spinal and cranial ganglia [9]. In *C. elegans*, the Mls-2 gene, a member of the HMX family, regulates cytoskeletal organization and cell elongation [26]. However, few contributions have been published about its mode of action in the eye. We therefore investigated its role in eye development.

We showed that HMX1 exerts an inhibitory effect on *EPHA6* and that dimerization is necessary for this activity. Luciferase assays are known for producing artefactual results. By increasing the number of replicates and analyzing only the experiments where all conditions showed similar transfection efficiencies, we were able to obtain stable results, which were further confirmed by ChIP and *in vivo* experiments in zebrafish. Mutations that removed the dimerization domains of HMX1, i.e. the HD and SD1 domains, abolished its inhibitory efficiency on *EPHA6* promoter. Removing the HD also perturbed the cellular localization of HMX1, which was no longer restricted to the nucleus. All other mutants, including deletion of the SD1 domain, maintained a strict nuclear expression indicating that SD1 is involved in dimerization while the HD is necessary both for dimerization and nuclear localization. In addition to ChIP validation in mouse retina, we also showed that ectopic overexpression of HMX1 in the whole eye in a zebrafish transgenic animal in which expression of HMX1 was under a heat-shock-inducible promoter was accompanied by a reduction of *epha4b* ocular expression, the zebrafish functional homolog of *EPHA6*.

The role of the SD2 domain remains unknown. We showed that a deletion mutant, which was dimerizing normally, was not inhibiting the *EPHA6* promoter like the wild type protein, but was slightly activating it, instead. This conserved domain could thus be an interaction site for a cofactor necessary for the inhibition action of HMX1.

When deleting the homeobox, we observed that the GFP-HMX1 fusion protein lost its specific nuclear localization, and became expressed in a punctate manner in the nucleus as well as in the cytoplasm. Our first hypothesis was that the protein lacking the homeobox was misfolded, and therefore activated clearance mechanisms, either by autophagy or by the proteasome degradation system. The homeobox of HMX1 has a well defined helix-loop-helix-turn-helix tertiary structure type. It is possible that deleting it entirely or part of it changes the three-dimensional structure enough to activate the clearance mechanisms for misfolded proteins. However, we could not detect any indication that these mechanisms were triggered. One of the main components of autophagosomes is LC3B, and we therefore tested whether it colocalized with GFP-HMX1 del HD. This was, however, not the case even after blocking autophagy using a chloroquine treatment. We did not observe an increase in expression of ubiquitin and p62, confirming that the HMX1 aggregates were not autophagosomes, and that the proteasome was not activated. The HMX1 del HD aggregates did not induce cell death either as we observed no increase in PI-stained cells compared to other transfections (not shown). Moreover, PARP was not cleaved by Caspase 3 in GFP-HMX1 del HD transfections, indicating an absence of apoptosis (not shown). Thus, we do not know at this time what is the exact nature of these GFP-HMX1 del HD aggregates and if they represent pure HMX1 aggregates or a more complex structure.

In a previous study, we showed that a morpholino-based knock-down of zebrafish *hmx1* had no effect on retinal patterning [11], which is in contradiction to the results obtained previously in the chick retina [10] and the results presented here. However, in chicken the relationship between HMX1 and EPHA3 was shown by overexpressing HMX1 on the temporal side of the retina where it is not expressed normally. The same procedure was used in the current work. In our previous study, *hmx1* was knocked-down on the nasal side of the retina, whereas the temporal part was unaffected by this procedure, and *epha4b* was able to play its role in the temporal retina.

In summary, we showed that HMX1 exerts its inhibitory activity through a dimer and identified *EPHA6* as a target of HMX1. Identifying other targets will allow us to further understand the role of HMX1.
